# Severe mental illness and mortality and coronary revascularisation following a myocardial infarction: a retrospective cohort study

**DOI:** 10.1186/s12916-021-01937-2

**Published:** 2021-03-22

**Authors:** Kelly Fleetwood, Sarah H. Wild, Daniel J. Smith, Stewart W. Mercer, Kirsty Licence, Cathie L. M. Sudlow, Caroline A. Jackson

**Affiliations:** 1 Usher Institute, University of Edinburgh, Teviot Place, Edinburgh, EH8 9AG UK; 2 Institute of Health and Wellbeing, University of Glasgow, Glasgow, UK; 3 Information Services Division, National Services Scotland, NHS Scotland, Edinburgh, UK

**Keywords:** Myocardial infarction, Schizophrenia, Bipolar disorder, Depression, Prognosis, Coronary revascularisation, Mortality

## Abstract

**Background:**

Severe mental illness (SMI), comprising schizophrenia, bipolar disorder and major depression, is associated with higher myocardial infarction (MI) mortality but lower coronary revascularisation rates. Previous studies have largely focused on schizophrenia, with limited information on bipolar disorder and major depression, long-term mortality or the effects of either sociodemographic factors or year of MI. We investigated the associations between SMI and MI prognosis and how these differed by age at MI, sex and year of MI.

**Methods:**

We conducted a national retrospective cohort study, including adults with a hospitalised MI in Scotland between 1991 and 2014. We ascertained previous history of schizophrenia, bipolar disorder and major depression from psychiatric and general hospital admission records. We used logistic regression to obtain odds ratios adjusted for sociodemographic factors for 30-day, 1-year and 5-year mortality, comparing people with each SMI to a comparison group without a prior hospital record for any mental health condition. We used Cox regression to analyse coronary revascularisation within 30 days, risk of further MI and further vascular events (MI or stroke). We investigated associations for interaction with age at MI, sex and year of MI.

**Results:**

Among 235,310 people with MI, 923 (0.4%) had schizophrenia, 642 (0.3%) had bipolar disorder and 6239 (2.7%) had major depression. SMI was associated with higher 30-day, 1-year and 5-year mortality and risk of further MI and stroke. Thirty-day mortality was higher for schizophrenia (OR 1.95, 95% CI 1.64–2.30), bipolar disorder (OR 1.53, 95% CI 1.26–1.86) and major depression (OR 1.31, 95% CI 1.23–1.40). Odds ratios for 1-year and 5-year mortality were larger for all three conditions. Revascularisation rates were lower in schizophrenia (HR 0.57, 95% CI 0.48–0.67), bipolar disorder (HR 0.69, 95% CI 0.56–0.85) and major depression (HR 0.78, 95% CI 0.73–0.83). Mortality and revascularisation disparities persisted from 1991 to 2014, with absolute mortality disparities more apparent for MIs that occurred around 70 years of age, the overall mean age of MI. Women with major depression had a greater reduction in revascularisation than men with major depression.

**Conclusions:**

There are sustained SMI disparities in MI intervention and prognosis. There is an urgent need to understand and tackle the reasons for these disparities.

**Supplementary Information:**

The online version contains supplementary material available at 10.1186/s12916-021-01937-2.

## Background

Cardiovascular disease (CVD) is a major contributor to the marked premature mortality in people with severe mental illness (SMI), including schizophrenia, bipolar disorder and major depression [1–5]. There has been considerable research on the impact of SMI on CVD occurrence, but far less on the effect on CVD outcomes. Over the past 20 years, accumulating evidence suggests that people with SMI have poorer outcomes following a myocardial infarction (MI) [6–8] and are less likely to receive coronary revascularisation than those without mental illness [7, 9–12]. However, most studies have examined the effect of schizophrenia on MI outcomes [8, 11], with far less investigation of the effect of prior bipolar disorder [6, 7, 9, 13] or major depression [7, 10]. Effects of SMI on longer-term mortality (beyond 1-year post-MI) have rarely been studied [14], no study (to our knowledge) has specifically assessed further MI and there are limited data on whether associations differ by age at MI and sex. The relative risk of mortality in younger people with schizophrenia may be lower than in older people [6], whilst women with SMI may be treated more conservatively than men with the same illness [7].

It is unclear whether disparities in mortality and revascularisation are changing over time. The MI mortality gap for schizophrenia compared to the general population may have widened between 1995 and 2015 in Denmark [14], whilst mental health disparities in revascularisation rates do not appear to be narrowing in the USA [9, 10]. To our knowledge, no study has reported trends in coronary revascularisation following MI by year of MI and SMI status in a universal healthcare setting.

We therefore conducted a retrospective cohort study to investigate the effect of a previous diagnosis of schizophrenia, bipolar disorder and major depression on risks of mortality, further MI and other vascular events, and rates of revascularisation following hospital admission for MI, by year of MI, age at MI and sex. We analysed SMI conditions separately to achieve greater epidemiological insight across these disorders. Whilst they share some commonalities in the pattern of association with physical disease, it may be inappropriate to assume they are homogeneous with respect to patterns of physical disease outcomes and receipt of clinical care.

## Methods

This study is reported in accordance with the Standard Reporting of Observational Studies in Epidemiology (STROBE) [15] and Reporting of Studies Conducted using Observational Routinely Collected Health Data (RECORD) [16] statements.

### Study population

We identified all adults aged 18 and over with a diagnosis of MI recorded in the Scottish Morbidity Record General/Acute Inpatient and Day Case dataset (SMR01, see https://www.ndc.scot.nhs.uk/Data-Dictionary/SMR-Datasets/SMR01-General-Acute-Inpatient-and-Day-Case/) between 1 January 1991 and 31 December 2014. SMR01 includes details of all acute hospital admissions in Scotland from 1980 onwards and information on clinical procedures/interventions coded using the fourth revision of the OPCS Classification of Interventions and Procedures (OPCS-4). We identified incident MIs using the ninth and tenth revisions of the International Classification of Diseases (ICD9 410 and ICD10 I21 and I22), where codes were included in a primary or secondary diagnosis field. We defined incident events as MIs that occurred between 1 January 1991 and 31 December 2014, where no previous admissions to hospital for MI had been recorded during the preceding 10 years.

### Definition and ascertainment of SMI

We determined the history of a mental health condition from the Scottish Mental Health Inpatient and Day Case (SMR04) dataset (which records psychiatric hospital admissions from 1981 onward) and the SMR01 dataset. We identified mental health conditions from primary or secondary diagnosis fields of admissions that occurred after the individual’s 18th birthday and before their incident MI (Additional file 1: Table S1) [17, 18]. We categorised people into three mutually exclusive SMI groups. Where individuals had a record of more than one diagnosis, we used a severity hierarchy, with schizophrenia considered the most severe, followed by bipolar disorder and major depression. We compared MI outcomes in people with a history of each of these three disorders to those with no prior hospital record for any mental health condition.

### Outcomes

Primary outcomes were 30-day and 1-year mortality and coronary revascularisation. Secondary outcomes included 5-year mortality, mortality over the entire follow-up period, time to further MI and time to further vascular event (fatal or non-fatal MI or stroke). We identified deaths up to 31 December 2018 from Scottish death records which include date and cause of death, as recorded on the death certificate. The analysis of 5-year mortality includes a smaller cohort of MI admissions up to 2013 so that all individuals potentially have at least 5 years of follow-up. For mortality over the entire follow-up period, time to death was calculated from the date of the incident MI to the date of death, with follow-up to the end of 2018. We identified the following coronary revascularisation procedures from OPCS-4 codes recorded in SMR01 (Additional file 1: Table S2) [19]: percutaneous transluminal coronary angioplasty (PTCA), percutaneous coronary intervention (PCI) and coronary artery bypass graft (CABG). We investigated coronary revascularisation within 30 days in line with the time frame used in previous studies of revascularisation following MI. Time to revascularisation was calculated from the date of the incident MI to the date of the first subsequent revascularisation procedure, regardless of whether the individual experienced another MI. We identified further events (occurring more than 30 days after the index MI) from SMR01 and death records based on conditions recorded in primary or secondary fields. We identified further MI using the same ICD-9 and ICD-10 codes as for the index MI and stroke using ICD-9 codes 430, 431, 434 and 436 and ICD-10 codes I60, I61, I63 and I64. Time to further MI and time to further vascular event were each calculated from the date of the incident MI to the date of the next MI or vascular event, as appropriate, regardless of whether revascularisation was received.

### Sociodemographic and clinical covariates

Area-based deprivation, urbanicity and NHS health board (with Scotland divided into 14 regional areas for the purposes of healthcare delivery) were defined based on each person’s place of residence at the time of MI. Area-based deprivation was measured by the Carstairs Index in line with recommendations for the analysis of deprivation in Scotland where the time frame starts prior to 1996 [20]. The Carstairs Index is based on four census variables (car ownership, male unemployment, household overcrowding and low occupational social class), calculated at the postcode sector level and divided into quintiles based on the entire Scottish population (Additional file 1: Text S1) [20–22]. Urbanicity was classified according to the Scottish Government sixfold urban rural indicator [23]. We ascertained history of alcohol use disorder based on all hospital records before the date of first MI and defined it using ICD codes for mental and behavioural disorders due to alcohol use and physical health conditions caused by alcohol use disorder (as listed in Additional file 1: Table S3). For each MI, we used ICD codes to identify diagnoses of diabetes, chronic obstructive pulmonary disease (COPD) and heart failure from the hospital discharge record for the index MI (Additional file 1: Table S4).

### Statistical analyses

We used direct standardisation to calculate sex-specific age-standardised rates for the three primary outcomes, by age at MI and year of MI group. We used only four age groups (< 60, 60–69, 70–79 and ≥ 80 years) and four year groups of equal duration to ensure that there were sufficient numbers within each group for the standardisation. We derived sex-specific age distributions for the standard population from the distributions observed in the period 2003–2008.

We used logistic regression to model 30-day, 1-year and 5-year mortality, and Cox proportional hazards regression for coronary revascularisation, mortality during the entire follow-up period and time to further events. For coronary revascularisation and time to further events, we accounted for death as a competing risk, censoring at the date of death. To evaluate coronary revascularisation within 30 days, we also censored individuals alive at 30 days at this time point. We included age at MI and year of MI as continuous variables modelled as fractional polynomials in order to allow for non-linear relationships between these variables and the outcomes [24], and the remaining covariates as categorical variables. For each outcome, model 1 included SMI, age at MI, sex and year of MI, while model 2 additionally included history of alcohol use disorder, deprivation, urbanicity and health board. To investigate whether associations between SMI and each of the outcomes varied by age at MI, sex and year of MI model 3 also included two-way interaction terms between mental health condition and each of these variables. Based on model 3, we used analysis of deviance to evaluate the statistical significance of the interactions and plotted odds ratios (or hazard ratios) by year of MI and sex for people with MI at different ages. We also developed a Shiny app to display additional interactive plots of trends by year of MI and age at MI (https://uiphsi-mdcvddm.shinyapps.io/mi-outcomes-mmd/). Among those who survived more than 30 days, we fitted a fourth model for analyses of mortality and further events, adjusting for receipt of revascularisation within 30 days.

In sensitivity analyses, we redefined major depression based on psychiatric hospital admission records only and identified coronary revascularisation within 90 days of MI. We used R version 3.6.1 for all analyses [25].

## Results

### Cohort characteristics

We identified 243,091 people with a first-ever MI between 1991 and 2014. After exclusions, we included 235,310 in our study cohort (see flow chart in Additional file 1: Fig. S1). Of these, 923 (0.4%), 642 (0.3%) and 6239 (2.7%) had a hospital admission record of schizophrenia, bipolar disorder and major depression, respectively. The prevalence of each SMI was higher in 2014 than in 1991 (0.6% versus 0.2% for schizophrenia, 0.4% versus 0.2% for bipolar disorder and 3.9% versus 1.7% for depression), which partly reflects a longer look-back period for hospital recording of SMI in people whose MI occurred in later years of the study period. Comparison of SMI prevalence using the minimum 10-year look-back period for all MI patients still revealed an increased prevalence of each SMI in later years, suggesting that improved diagnosis of SMI over time and better recording of SMI in general hospital admission records might also play a role. Almost 60% of the cohort were male. There were more men than women in the group with schizophrenia, but more women among those with bipolar disorder and major depression (Table 1). MI occurred at a younger age in people with schizophrenia and bipolar disorder (62 and 68 years, respectively) than those with no mental health condition (69 years). Compared to those with no mental illness, people with each psychiatric disorder tended to be of lower socioeconomic status, more likely to have an alcohol use disorder and more likely to have diabetes, COPD and heart failure recorded in their MI admission record. The median follow-up time was 5.3 years (interquartile range 0.4 to 11.6). Overall, 30-day and 1-year mortality were 19.7% and 29.7%, respectively, and 16.4% received coronary revascularisation within 30 days (Table 1, Additional file 1: Tables S5 and S6).

**Table 1:** Baseline characteristics and outcomes of patients with MI, by a history of mental health admission

	No mental health admission (*N* = 227,506)	Schizophrenia (*N* = 923)	Bipolar disorder (*N* = 642)	Depression (*N* = 6239)
Median follow-up time (IQR), years	5.4 (0.4, 11.7)	4.3 (0.2, 8.9)	3.2 (0.1, 8.6)	3.2 (0.1, 8.0)
Sex, *n* (%)				
Female	93,813 (41.2%)	369 (40.0%)	347 (54.0%)	3555 (57.0%)
Male	133,693 (58.8%)	554 (60.0%)	295 (46.0%)	2684 (43.0%)
Mean age at MI (SD), years	69.1 (13.2)	62.3 (13.5)	67.5 (12.7)	68.9 (13.8)
Year of MI admission, *n* (%)				
1991–1995	60,050 (26.4%)	165 (17.9%)	121 (18.8%)	1020 (16.3%)
1996–2000	48,701 (21.4%)	168 (18.2%)	106 (16.5%)	1067 (17.1%)
2001–2005	44,426 (19.5%)	196 (21.2%)	161 (25.1%)	1278 (20.5%)
2006–2010	39,507 (17.4%)	184 (19.9%)	135 (21.0%)	1461 (23.4%)
2011–2014	34,822 (15.3%)	210 (22.8%)	119 (18.5%)	1413 (22.6%)
Deprivation quintile, *n* (%)				
1 (most deprived)	50,898 (22.4%)	279 (30.2%)	156 (24.3%)	1609 (25.8%)
2	47,691 (21.0%)	195 (21.1%)	133 (20.7%)	1345 (21.6%)
3	45,792 (20.1%)	188 (20.4%)	106 (16.5%)	1213 (19.4%)
4	44,557 (19.6%)	163 (17.7%)	125 (19.5%)	1131 (18.1%)
5 (least deprived)	38,568 (17.0%)	98 (10.6%)	122 (19.0%)	941 (15.1%)
Urbanicity, *n* (%)				
Large urban area	78,299 (34.4%)	365 (39.5%)	271 (42.2%)	2408 (38.6%)
Other urban area	80,536 (35.4%)	354 (38.4%)	203 (31.6%)	2064 (33.1%)
Accessible small town	20,597 (9.1%)	53 (5.7%)	49 (7.6%)	527 (8.4%)
Remote small town	9415 (4.1%)	28 (3.0%)	29 (4.5%)	291 (4.7%)
Accessible rural	23,960 (10.5%)	84 (9.1%)	56 (8.7%)	532 (8.5%)
Remote rural	14,699 (6.5%)	39 (4.2%)	34 (5.3%)	417 (6.7%)
History of alcohol use disorder, *n* (%)	5177 (2.3%)	138 (15.0%)	70 (10.9%)	915 (14.7%)
Diabetes recorded at MI admission, *n* (%)	22,449 (9.9%)	115 (12.5%)	74 (11.5%)	772 (12.4%)
COPD recorded at MI admission, *n* (%)	12,523 (5.5%)	77 (8.3%)	45 (7.0%)	659 (10.6%)
Heart failure at MI admission, n (%)	37,796 (16.6%)	171 (18.5%)	110 (17.1%)	1067 (17.1%)
30-day mortality, *n* (%)	44,580 (19.6%)	203 (22.0%)	154 (24.0%)	1443 (23.1%)
1-year mortality, *n* (%)	66,977 (29.4%)	309 (33.5%)	247 (38.5%)	2306 (37.0%)
Revascularisation within 30 days*				
Any, *n* (%)	37,375 (16.4%)	137 (14.8%)	94 (14.6%)	1036 (16.6%)
CABG, *n* (%)	3175 (1.4%)	7 (0.8%)	8 (1.2%)	56 (0.9%)
PTCA, *n* (%)	8219 (3.6%)	30 (3.3%)	22 (3.4%)	193 (3.1%)
PCI, *n* (%)	26,450 (11.6%)	104 (11.3%)	65 (10.1%)	805 (12.9%)
5-year mortality^†^				
*N*	219,161	868	611	5897
*n* (%)	100,560 (45.9%)	457 (52.6%)	350 (57.3%)	3388 (57.5%)

*CABG* coronary artery bypass graft, *COPD* chronic obstructive pulmonary disease, *PCI* percutaneous coronary intervention, *PTCA* percutaneous transluminal coronary angioplasty

*Individuals may have received more than one type of revascularisation operation

^†^Based on the 225,730 individuals with their first MI between 1991 and 2013

### Absolute rates of mortality and coronary revascularisation by time period

**Fig. 1:**
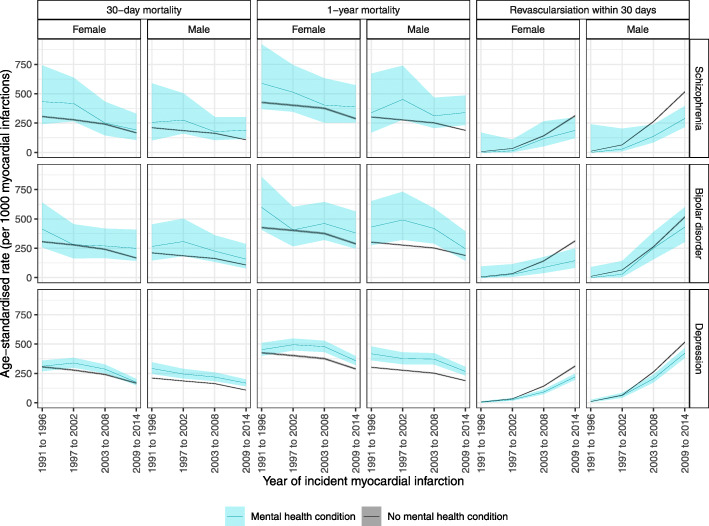
Age-standardised rates of 30-day mortality, 1-year mortality and coronary revascularisation within 30 days following a myocardial infarction, by previous hospitalisation with severe mental illness, 1991–2014. Shading represents 95% confidence intervals

In general, absolute age-standardised sex-specific proportions of 30-day and 1-year mortality were higher in the groups with a SMI identified from hospital records than in the comparison population with no such record and declined between 1991 and 2014 in all groups (Fig. 1). However, smaller numbers of people with schizophrenia and bipolar disorder introduce a greater degree of uncertainty in the pattern of change over time for these conditions. Whilst the absolute proportion of coronary revascularisation within 30 days increased over time in all comparison groups, rates remained much lower in people with each psychiatric disorder than in the comparison group.

### Relative effect of SMI on MI outcomes and coronary revascularisation

After adjusting for age at MI, year of MI and sex, having a prior hospitalisation record for each of schizophrenia, bipolar disorder and major depression was associated with statistically significant higher risks of mortality and further events, but lower rates of coronary revascularisation. These associations attenuated slightly after additional adjustment for alcohol use disorder, deprivation, urbanicity and health board (Table 2; model 2). The odds of 30-day mortality were higher in people with schizophrenia (OR 1.95, 95% CI 1.64–2.30), bipolar disorder (OR 1.53, 95% CI 1.26–1.86) and depression (OR 1.31, 95% CI 1.23–1.40) compared to the group with no hospital record of a mental health condition. There were larger differences in 1-year and 5-year mortality (Table 2). Among those who survived the first 30 days post-MI, a prior history of each SMI was associated with increased odds of over a third of further MI and further vascular events (Table 2). All three disorders were associated with a substantially reduced rate of coronary revascularisation within 30 days, which was most marked for schizophrenia (HR 0.57, 95% CI 0.48–0.67; Table 2). Sensitivity analyses for each of the outcomes gave similar results (Additional file 1: Table S7).

**Table 2:** Effect estimates for MI outcomes in people with versus without a prior mental health admission

Outcome	Number	Model	Schizophrenia	Bipolar disorder	Depression
30-day mortality, OR (95% CI)	235,310	Model 1	2.06 (1.74, 2.44)	1.58 (1.30, 1.91)	1.37 (1.29, 1.46)
		Model 2	1.95 (1.64, 2.30)	1.53 (1.26, 1.86)	1.31 (1.23, 1.40)
1-year mortality, OR (95% CI)	235,310	Model 1	2.41 (2.07, 2.81)	1.99 (1.66, 2.37)	1.65 (1.55, 1.75)
		Model 2	2.22 (1.91, 2.59)	1.90 (1.59, 2.27)	1.53 (1.45, 1.63)
5-year mortality, OR (95% CI)	226,537*	Model 1	3.11 (2.67, 3.63)	2.28 (1.89, 2.75)	2.08 (1.96, 2.22)
		Model 2	2.75 (2.35, 3.20)	2.15 (1.78, 2.59)	1.82 (1.71, 1.94)
Mortality during follow-up, HR (95% CI)	235,310	Model 1	1.97 (1.82, 2.12)	1.63 (1.50, 1.79)	1.44 (1.40, 1.49)
		Model 2	1.82 (1.68, 1.96)	1.55 (1.42, 1.70)	1.35 (1.31, 1.39)
Time to further MI, HR (95% CI)	188,930^†^	Model 1	1.50 (1.31, 1.72)	1.38 (1.17, 1.63)	1.45 (1.38, 1.53)
		Model 2	1.42 (1.24, 1.63)	1.34 (1.13, 1.58)	1.38 (1.31, 1.45)
Time to further vascular event, HR (95% CI)	188,930^†^	Model 1	1.55 (1.37, 1.75)	1.45 (1.25, 1.68)	1.48 (1.41, 1.55)
		Model 2	1.46 (1.29, 1.65)	1.40 (1.20, 1.62)	1.40 (1.33, 1.46)
Revascularisation within 30 days, HR (95% CI)	235,310	Model 1	0.52 (0.44, 0.62)	0.66 (0.54, 0.80)	0.71 (0.67, 0.76)
		Model 2	0.57 (0.48, 0.67)	0.69 (0.56, 0.85)	0.78 (0.73, 0.83)

Model 1 is adjusted for age at MI, sex and year of MI. Model 2 is adjusted for age at MI, sex, year of MI, history of alcohol use disorder, deprivation, urbanicity and health board

*HR* hazard ratio, *OR* odds ratio

*Myocardial infarction admissions up to 2013 in order to ensure that all individuals have at least 5 years of follow-up

^†^Individuals who survived more than 30 days after MI

### Interaction between SMI and year of MI, sex and age at MI

**Fig. 2:**
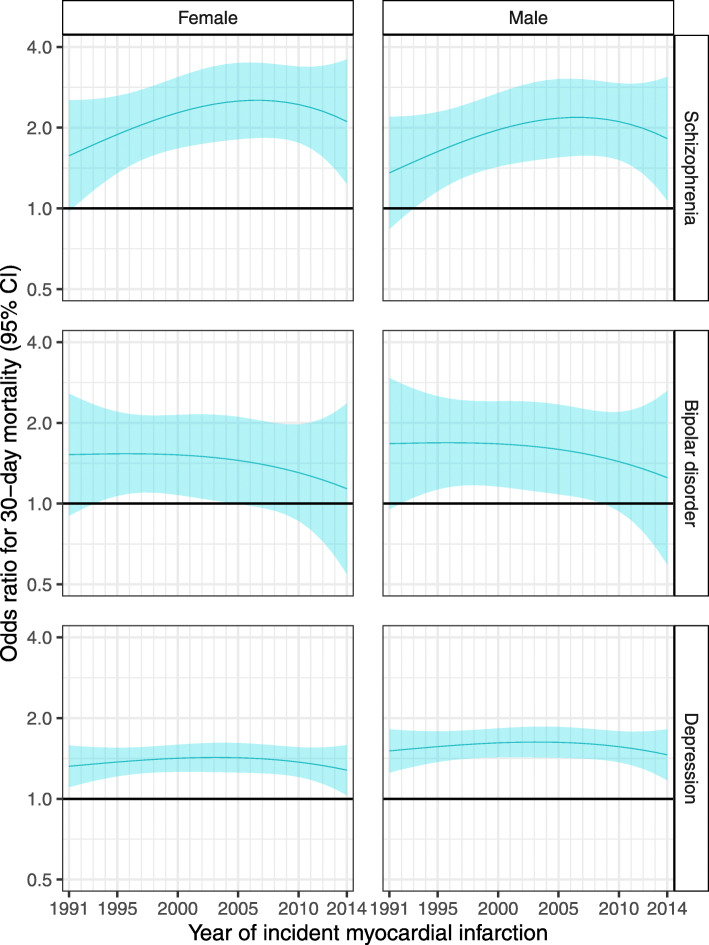
Sex-specific odds ratios and 95% confidence intervals for 30-day mortality following a myocardial infarction, among 70-year-olds, comparing people with a prior hospital record for each SMI versus no prior record of any mental health condition, 1991–2014. Estimates were obtained from a logistic regression model adjusting for age at MI, year of MI, sex, deprivation, urbanity, health board and history of an alcohol use disorder, including interactions between SMI and each of age at MI, year of MI and sex. Shading represents 95% confidence intervals

**Fig. 3:**
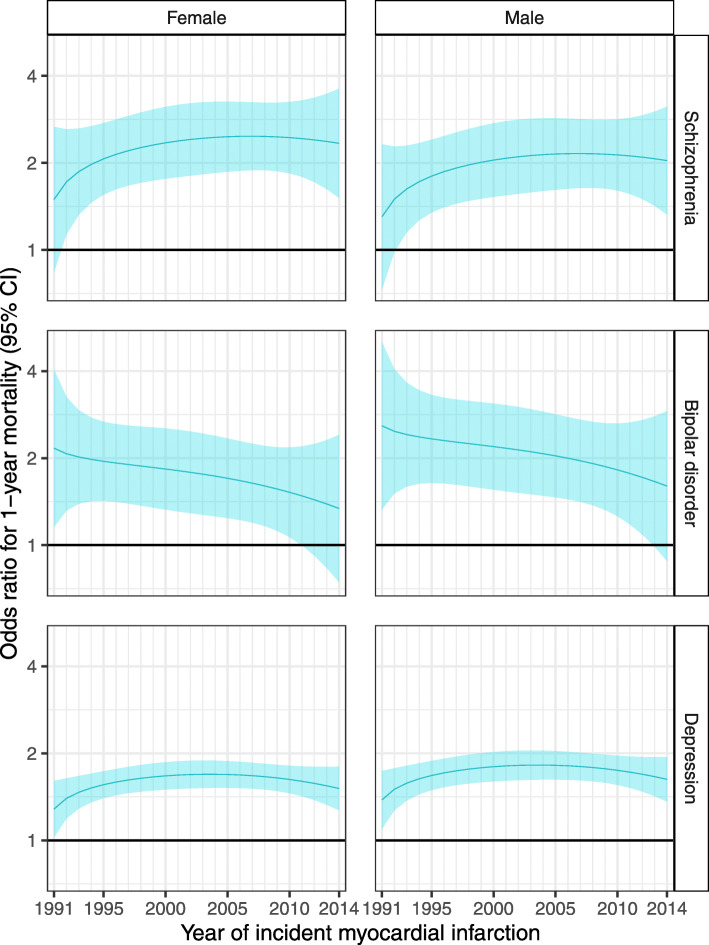
Sex-specific odds ratios and 95% confidence intervals for 1-year mortality following a myocardial infarction, among 70-year-olds, comparing people with a prior hospital record for each SMI versus no prior record of any mental health condition, 1991–2014. Estimates were obtained from a logistic regression model adjusting for age at MI, year of MI, sex, deprivation, urbanity, health board and history of an alcohol use disorder, including interactions between SMI and each of age at MI, year of MI and sex. Shading represents 95% confidence intervals

**Fig. 4:**
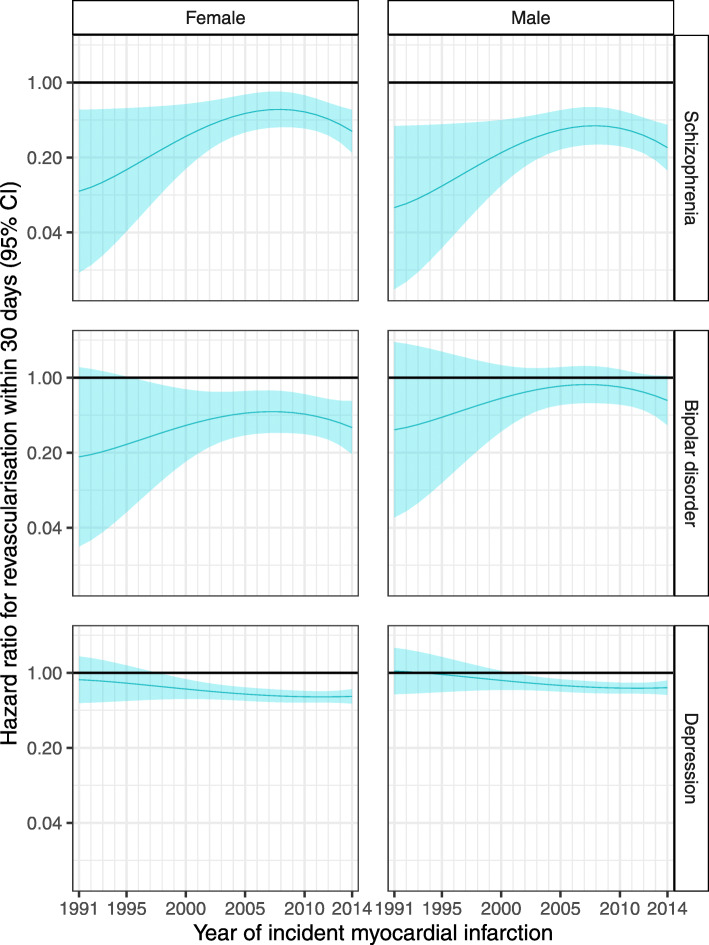
Sex-specific hazard ratios and 95% confidence intervals for revascularisation within 30 days following a myocardial infarction, among 70-year-olds, comparing people with a prior hospital record for each SMI versus no prior record of any mental health condition, 1991–2014. Estimates were obtained from a Cox proportional hazards model adjusting for age at MI, year of MI, sex, deprivation, urbanity, health board and history of an alcohol use disorder, including interactions between SMI and each of age at MI, year of MI and sex. Shading represents 95% confidence intervals

From 1991 to 2014, there was no clear change in the odds ratios for 30-day, 1-year and 5-year mortality (Figs. 2 and 3, Additional file 1: Table S8 and Fig. S2). However, survival analysis for the whole follow-up period (which has more statistical power than logistic regression) suggested that for MIs that occurred in later years, there was a widening of the disparity between people with a hospitalisation record of major depression and those with no hospital record for any mental health condition (Additional file 1: Table S8 and Fig. S3). For MIs occurring at age 70 (approximately the mean age at first MI), the hazard ratio for death during follow-up for individuals with major depression versus those without a mental health condition increased from 1.01 (95% CI 0.81–1.26) for MIs in 1991 to 1.43 (95% CI 1.29–1.58) for MIs in 2014 in women and from 1.07 (95% CI 0.86–1.34) to 1.52 (95% CI 1.37–1.69) in men. We observed a similar relative percentage increase in the hazard ratios for other ages. The results for people with schizophrenia and bipolar disorder were uncertain, with wide confidence intervals, due to smaller numbers of people in these groups. There was no evidence of a change from 1991 to 2014 in the association between SMI and revascularisation (Fig. 4, Additional file 1: Table S8 and Fig. S6). Again, results for people with schizophrenia and bipolar disorder were uncertain.

There was no statistically significant interaction between SMI and sex for the mortality outcomes, but the reduction in revascularisation among those with major depression was greater for women than for men (Fig. 4 and Additional file 1: Fig. S6). There was an interaction between age at MI and SMI, for 30-day and 1-year mortality, mortality over the whole follow-up period and revascularisation (Additional file 1: Table S8, Shiny app). Absolute differences in mortality outcomes between those with and without SMI were generally greatest for MIs occurring between 60 and 80 years of age and smallest among younger ages.

Finally, among those surviving 30 days, adjusting for receipt of coronary revascularisation had minimal impact on the associations of SMI with mortality and further events.

## Discussion

For people with a MI, having a prior hospitalisation record of schizophrenia, bipolar disorder or major depression was associated with increased risk of dying in the short- and long term and having a further MI or another vascular event. People with a SMI were far less likely to receive coronary revascularisation; among those with pre-existing major depression, this effect was more marked in women than men. SMI disparities in both risks of death and receipt of revascularisation did not improve over the period from 1991 to 2014.

Our study benefits from several strengths. It is one of the largest to examine the effect of SMI on MI outcomes, and coronary revascularisation treatment. Our national cohort was representative of people admitted to hospital with an MI. We included people with bipolar disorder and major depression, who have previously been little studied in this context, as well as those with schizophrenia. Including only people with a hospital record of schizophrenia, bipolar disorder or depression identified individuals at the more severe end of the mental illness spectrum. Scotland has a universal healthcare system, and so our findings are unbiased by inequalities in access to care based on health insurance provision. The long period of data collection facilitated analysis of time trends in associations with mortality and revascularisation, which have previously been little studied. Moreover, we also investigated interaction by age at MI and sex.

Since we ascertained prior SMI from general and psychiatric hospital admission data, our findings may not be generalizable to people with a SMI for which they have never been admitted to hospital. There may have been selection bias in that people with less severe depression may have been included in the depression group because they were admitted to a general hospital for an unrelated disease. Reassuringly, sensitivity analyses where we defined depression based on psychiatric hospital admissions only gave very similar effect estimates. Unfortunately, we did not have information on the date of onset, or clinical diagnosis, of SMI (which may have occurred prior to the hospital admission record used to ascertain the presence of SMI) and so could not investigate the effect of duration of mental illness on MI prognosis. The lack of appropriate data meant that we were unable to account for potential confounding by lifestyle factors (other than alcohol use disorders), such as smoking, diet or physical exercise, or key comorbidities prior to hospital admission with MI. Interestingly, adjustment for these in previous studies did not appear to influence the effect of SMI on mortality and revascularisation [7, 9, 10, 12, 14]. The main focus of our study was on MI prognosis in terms of death, irrespective of cause, occurring at key clinical time points, and so comparison of death from specific causes, including CVD, was beyond the scope of this study. Similarly, whilst we explored whether differences in receipt of timely revascularisation partly explained observed disparities in outcomes, it was beyond the remit of this study to investigate more broadly the contribution of coronary revascularisation at any time point to these disparities.

The prevalence of each SMI condition among people hospitalised for MI in our study is highly consistent with that reported in a previous study that used a similar method of ascertaining SMI comorbidity [6]. The increased risks of short-term mortality among people with schizophrenia in our study are consistent with the findings in most previous studies [6, 7, 9, 12, 14, 26–28]. One study reported no difference in 30-day mortality between those with and without schizophrenia, but included far fewer people with schizophrenia than in our study [29]. The similarly increased risks of 30-day and 1-year mortality among people with bipolar disorder in our study are consistent with the findings from a Swedish study [6]. However, our findings on short-term mortality in those with both bipolar disorder and depression are not in keeping with other studies, which report either no difference in [7, 9, 12] or a reduced risk of [10] mortality in the former groups. However, potential limitations of these studies include reduced statistical power due to smaller numbers of patients with bipolar disorder [13] and reliance on MI admission records to determine the history of SMI potentially leading to under-ascertainment of history of bipolar disorder and depression [7, 9, 10]. We are aware of just one other study examining long-term prognosis after MI in the context of SMI, which also found that schizophrenia was associated with a marked increase in long-term mortality [14]. Our finding of interaction by age, but not sex, aligns with the findings from Bodén et al. [6]. The lack of interaction by sex on in-hospital mortality among those with major depression concurs with the post hoc findings from Schulman-Marcus et al. [10], but not with those from Mohammed et al., who reported a greater effect of depression (but not schizophrenia or bipolar disorder) on in-hospital MI mortality among men than among women [7]. Comparison of our findings on the risk of further events with previous studies is difficult, since these have either included further events within a composite outcome or analysed these as in-hospital complications only.

The magnitude of the effect of schizophrenia on receipt of coronary revascularisation in our study is in keeping with that reported in other studies similarly unselected for age [7, 9, 12, 27, 28]. Two previous studies similarly reported lower revascularisation rates among those with bipolar disorder [12] and depression [10]. As observed in our study, women in general are less likely than men to receive coronary revascularisation, with two studies also reporting that this was more marked among people with depression [7, 10]. The reasons for the latter observation are unclear and worthy of further investigation.

Despite advances in primary and secondary prevention of MI and overall improvement in MI mortality over time, our study reveals persistent SMI disparities in MI prognosis and coronary revascularisation over time in Scotland. A nationwide Danish study reported a widening of the gap in MI mortality between those with and without schizophrenia during a similar period as in our study [14]. Two US-based studies reported that the gap in revascularisation rates between those with and without these three psychiatric disorders persisted between 2002 and 2013 [9, 10]. Similarly, a recent Danish study reported no change over time in the association between schizophrenia and lower revascularisation rates among people with acute coronary syndrome (encompassing both MI and unstable angina) [30].

The reasons for poorer MI outcomes among people with a SMI remain unclear. The excess risks of mortality in the short- and long term and of further events in our study support a multifactorial explanation. Poorer lifestyle and comorbidity profiles in people with SMI do not appear to account for the increased mortality risk, and neither does MI severity [7, 9, 10, 12, 14]. Differences in acute intervention strategies could reflect reduced patient advocacy and capacity for procedural consent in those with psychiatric illness, physician concerns about compliance with medication and regular follow-up, and assumptions about lifestyle behaviour change. People with schizophrenia may be less likely to be offered investigation and treatment for MI and more likely to decline investigation and treatment [31]. However, robust qualitative studies are needed to identify patient- and physician-specific factors that may be contributing to the sub-optimal care of these vulnerable groups. Adjustment for receipt of coronary interventions only slightly attenuated mortality estimates in our study and in others [6, 13, 28], and poorer outcomes are still observed in those with psychiatric illness where the study population includes only those receiving coronary revascularisation [32].

Differences in the prescription of and adherence to pharmacological and lifestyle approaches to secondary prevention (critical to reducing MI mortality) could partly explain the apparent poorer effect of coronary revascularisation on outcomes and the increased risk of further events in those with SMI. A recent novel study comparing secondary prevention treatment reported higher mortality among untreated patients with schizophrenia compared to the treated general population, but no difference in mortality among those treated with any combination of triple therapy drugs [33]. Further study of this and other aspects of the MI care pathway in relation to psychiatric illness status is needed. Time from symptom onset to hospital arrival (onset-to-door) and from arrival to revascularisation (door-to-balloon) have been linked to 1-year mortality [34] and could differ by mental illness status. Cardiac rehabilitation is associated with reduced MI mortality, but multiple barriers, including low referral rates of patients from low socioeconomic groups and low participation due to psychological well-being [35], place people with SMI at a disadvantage in terms of accessing this. Finally, some antipsychotic and anti-depressant drugs prolong the Q-T interval [36] (an independent predictor of MI mortality [37]), and polypharmacy may augment this risk [38].

## Conclusions

We found sustained SMI disparities in MI intervention and prognosis. There is an urgent need to understand and tackle the reasons for these disparities. Meanwhile, cardiologists and general practitioners/family physicians should be acutely aware of the potential for under-treatment of MI patients who have a pre-existing SMI and ensure that clinical decision-making is not inappropriately influenced by these comorbidities. Improved inter-specialty collaboration, particularly between cardiologists and psychiatrists, and including effective communication with primary care could help minimise inequalities in clinical care and ultimately contribute to improved clinical outcomes for people with SMI.

## Supplementary Information

**Additional file 1: **: **Table S1.** ICD-9 and ICD-10 codes used to identify mental health conditions. **Table S2.** OPCS-4 codes used to identify coronary revascularisation procedures. **Text S1.** Carstairs Index. **Table S3.** ICD-9 and ICD-10 codes used to identify alcohol use disorder. **Table S4.** ICD codes used to identify comorbidities recorded during the incident MI admission. **Fig. S1.** Flow diagram for establishing the cohort. **Table S5.** Number of individuals and events per group. **Table S6.** Number of individuals and events per group – sensitivity analysis (depression based on psychiatric hospital admission records only). **Table S7.** Sensitivity analysis for models 1 and 2 (depression based on psychiatric hospital admission records only). **Table S8.** *P*-values for analysis of deviance comparing models without each interaction to the model including all three interactions. **Fig. S2.** Odds ratios for 5-year mortality comparing people with a hospital record for each SMI versus no record of any mental health condition. **Fig. S3.** Hazard ratios for mortality during follow-up comparing people with a hospital record for each SMI versus no record of any mental health condition. **Fig. S4.** Hazard ratios for time to further myocardial infarction comparing people with a hospital record for each SMI versus no record of any mental health condition. **Fig. S5.** Hazard ratios for time to further vascular event (MI or stroke) comparing people with a hospital record for each SMI versus no record of any mental health condition. **Fig. S6.** Hazard ratios for revascularisation within 90 days comparing people with a hospital record for each SMI versus no record of any mental health condition.

## Data Availability

The data that support the findings of this study are available from the National Services Scotland, but restrictions apply to the availability of these data, which are accessible to accredited researchers through application to the NHS Scotland Public Benefit and Privacy Panel for Health and Social Care and, upon approval, via access to the NHS National Safe Haven. The data underlying this article cannot be shared publicly.
