# Supplementary material for: Severe mental illness and mortality and coronary revascularisation following a myocardial infarction: a retrospective cohort study
Source: BMC Med. 2021 Mar 22;19:67. doi: 10.1186/s12916-021-01937-2 (PMC7983231; doi:10.1186/s12916-021-01937-2)

**Additional file 1**

**Severe mental illness and mortality and coronary revascularisation following a myocardial infarction: a retrospective cohort study**

Kelly Fleetwood^1^, Sarah H Wild^1^, Daniel J Smith^2^, Stewart W Mercer^1^, Kirsty Licence^3^, Cathie LM Sudlow^1^, Caroline A Jackson^1*^

^1^Usher Institute, University of Edinburgh

^2^Institute of Health and Wellbeing, University of Glasgow

^3^Information Services Division, National Services Scotland, NHS Scotland

*corresponding author:

Caroline Jackson

Usher Institute

University of Edinburgh

Teviot Place, Edinburgh EH8 9AG

Email: [caroline.jackson@ed.ac.uk](mailto:caroline.jackson@ed.ac.uk)

Tel: +44 (0)131 6503223

**Contents**

[Table S1: ICD-9 and ICD-10 codes used to identify mental health conditions 1](#_Toc59182858)

[Table S2: OPCS-4 codes used to identify coronary revascularisation procedures 2](#_Toc59182859)

[Text S1: Carstairs Index 3](#_Toc59182860)

[Table S3: ICD-9 and ICD-10 codes used to identify alcohol use disorder 4](#_Toc59182861)

[Table S4: ICD codes used to identify comorbidities recorded during the incident MI admission 5](#_Toc59182862)

[Figure S1: Flow diagram for establishing the cohort 6](#_Toc59182863)

[Table S5: Number of individuals and events per group 7](#_Toc59182864)

[Table S6: Number of individuals and events per group – sensitivity analysis (depression based on psychiatric hospital admission records only) 8](#_Toc59182865)

[Table S7: Sensitivity analysis for models 1 and 2 (depression based on psychiatric hospital admission records only) 9](#_Toc59182866)

[Table S8: P-values for analysis of deviance comparing models without each interaction to the model including all three interactions 11](#_Toc59182867)

[Figure S2: Odds ratios for 5-year mortality comparing people with a hospital record for each SMI versus no record of any mental health condition 12](#_Toc59182868)

[Figure S3: Hazard ratios for mortality during follow-up comparing people with a hospital record for each SMI versus no record of any mental health condition 13](#_Toc59182869)

[Figure S4: Hazard ratios for time to further myocardial infarction comparing people with a hospital record for each SMI versus no record of any mental health condition 14](#_Toc59182870)

[Figure S5: Hazard ratios for time to further vascular event (MI or stroke) comparing people with a hospital record for each SMI versus no record of any mental health condition 15](#_Toc59182871)

[Figure S6: Hazard ratios for revascularisation within 90 days comparing people with a hospital record for each SMI versus no record of any mental health condition 16](#_Toc59182872)

[References for the Supplementary Material 17](#_Toc59182873)

# Table S1: ICD-9 and ICD-10 codes used to identify mental health conditions

| **Mental health condition^1^** | **ICD10 codes^a^**  **(first 3 digits)** | **ICD9 codes^b^**  **(first 4 digits)** |
| --- | --- | --- |
| Schizophrenia: schizophrenia and schizoaffective disorders | F20, F25 | 295.0-295.3,  295.6-295.9 |
| Other psychoses: schizotypal disorders, acute and transient psychosis, delusional disorders, and other psychotic disorders | F21-F24,  F28, F29 | 295.4, 295.5,  297.0-297.9  298.3, 298.4, 298.8, 298.9 |
| Bipolar disorder: manic episode or bipolar affective disorder | F30-F31 | 296.0  296.2-296.6 |
| Depression: depressive episode or recurrent depressive disorder | F32-F33 | 296.1  298.0, 300.4, 311 |
| Other mental health conditions: including other mood disorders, neuroses, dissociative disorders, somatoform disorders, eating disorders, non-organic sleep disorders and other behavioural syndromes associated with physiological disturbances and physical factors, disorders of adult personality and behaviour, disorders of psychological development, behavioural and emotional disorders with onset in childhood and adolescence and unspecified mental disorders | F34-F69,  F80-F99 | 293.8,  296.8, 296.9,  298.1, 298.2,  299.0-301.9,  302.1-302.9,  305.9,  306.0-309.9,  312.0-315.9  316 |

a Further details on these codes can be found on the ICD-10 website [17].

b Further details on these codes can be found in the ICD-9 book [18].

# Table S2: OPCS-4 codes used to identify coronary revascularisation procedures

| Procedure type | OPCS-4 code | Definition |
| --- | --- | --- |
| Coronary artery bypass graft | K40 | Saphenous vein graft replacement of coronary artery |
|  | K41 | Other autograft replacement of coronary artery |
|  | K42 | Allograft replacement of coronary artery |
|  | K43 | Prosthetic replacement of coronary artery |
|  | K44 | Other replacement of coronary artery |
|  | K45 | Connection of thoracic artery to coronary artery |
|  | K46 | Other bypass of coronary artery |
| Percutaneous transluminal coronary angioplasty | K49 | Transluminal balloon angioplasty of coronary artery |
|  | K50.1 | Percutaneous transluminal laser coronary angioplasty |
|  | K50.8 | Other specified other therapeutic transluminal operations on coronary artery^1^ |
| Percutaneous coronary intervention | K75 | Percutaneous transluminal balloon angioplasty and insertion of stent into coronary artery |

1. Glasgow Western Infirmary pre April 2002 only, as per National Statistics publications that report revascularisation procedures in Scotland [19].

# Text S1: Carstairs Index

The Carstairs Index is based on four census variables (car ownership, male unemployment, household overcrowding and low occupational social class) [21]. It is calculated at the postcode sector level, where each postcode sector includes approximately 5,000 people and divided into quintiles based on the entire Scottish population [21]. The 2011 Carstairs Index defined the four variables as follows [21]:

- No car ownership: the number of people living in private households without a car divided by the total number of people living in private households.
- Male unemployment: the number of economically active males seeking or waiting to start work divided by the total number of all economically active males.
- Overcrowding: the number of people living in private households with more than one person per room divided by the total number of people living in private households.
- Low social class: the number of people living in private households where the household reference person (defined on the basis of working pattern, e.g. full-time or part-time, and age [22]) is economically active and in a low social class based on their occupation divided by the total number of people living in private households with an economically active household reference person.

Earlier versions of the Carstairs Index used broadly similar definitions of the four variables, with some differences in how overcrowding and low social class were defined between the versions [21].

The Carstairs Index for each postcode sector is calculated by standardizing each of the four variables by its Scotland wide mean and standard deviation, and then summing the four standardized values [21].

Following recommendations for the analysis of deprivation in Scotland [20], we used the following releases of the Carstairs Index, according to the date of the incident myocardial infarction.

Table: Recommended Carstairs Index release by date of incident myocardial infarction

| Date of incident myocardial infarction | Carstairs Index release |
| --- | --- |
| 1991 – 1996 | 1991 |
| 1997 – 2006 | 2001 |
| 2007 - 2014 | 2011 |

# Table S3: ICD-9 and ICD-10 codes used to identify alcohol use disorder

| ICD-10 Code | Description | ICD-9 Code | Description | |  |
| --- | --- | --- | --- | --- | --- |
| **Mental & behavioural disorders due to use of alcohol** | |  | |  |  |
| F10.1  F10.2  F10.3  F10.4  F10.6 | Harmful use  Dependence syndrome  Withdrawal state  Withdrawal state with delirium  Amnesic syndrome | 291.0  291.1  291.2  291.5  303 | Delirium tremens  Korsakov's psychosis, alcoholic  Other alcoholic dementia  Alcoholic jealousy  Alcohol dependence syndrome | |  |
| **Alcoholic liver disease** | |  | |  |  |
| K70.0  K70.1  K70.2  K70.3  K70.4  K70.9 | Alcoholic fatty liver  Alcoholic hepatitis  Alcoholic fibrosis and sclerosis of liver  Alcoholic cirrhosis of liver  Alcoholic hepatic failure  Alcoholic liver disease, unspecified | 571.0  571.1  571.2  571.3 | Alcoholic fatty liver  Acute alcoholic hepatitis  Alcoholic cirrhosis of liver  Alcoholic liver damage, unspecified | |  |
| **Other conditions** | | | | | |
| E24.4 | Alcohol induced Pseudo-Cushing’s syndrome |  | No equivalent code in ICD-9 | |  |
| E51.2 | Wernicke’s Encephalopathy |  | No equivalent code in ICD-9 | |  |
| G31.2 | Degeneration of nervous system due to alcohol |  | No equivalent alcohol-specific code included in ICD-9 | |  |
| G62.1 | Alcoholic polyneuropathy | 357.5 | Alcoholic polyneuropathy | |  |
| G72.1 | Alcoholic myopathy |  | No equivalent alcohol-specific code included in ICD-9 | |  |
| I42.6 | Alcoholic cardiomyopathy | 425.5 | Alcoholic cardiomyopathy | |  |
| K29.2 | Alcoholic gastritis | 535.3 | Alcoholic gastritis | |  |
| K85.2 | Alcohol-induced acute pancreatitis |  | No equivalent alcohol-specific code included in ICD-9 | |  |
| K86.0 | Alcohol-induced chronic pancreatitis |  | No equivalent alcohol-specific code included in ICD-9 | |  |
| O35.4 | Maternal care for (suspected) damage to foetus from alcohol |  | No equivalent alcohol-specific code included in ICD-9 | |  |
| Y57.3 | Drugs, medicaments and biological substances causing adverse effects in therapeutic use: alcohol deterrents | E947.3 | Drugs, medicaments and biological substances causing adverse effects in therapeutic use: alcohol deterrents | |  |
| Z50.2 | Alcohol rehabilitation |  | No equivalent alcohol-specific code included in ICD-9 | |  |
| Z71.4 | Alcohol abuse counselling and surveillance |  | No equivalent alcohol-specific code included in ICD-9 | |  |

# Table S4: ICD codes used to identify comorbidities recorded during the incident MI admission

| **Comorbidity** | **ICD10 codes**  **(first 3 digits)** | **ICD9 codes**  **(first 3 digits)** |
| --- | --- | --- |
| Diabetes | E10-14 | 250 |
| Chronic obstructive pulmonary disease | J40-J44 | 490-492, 496 |
| Heart failure | I50 | 428 |

# Figure S1: Flow diagram for establishing the cohort

**SMR01 records for adults**

**(1991-2014)**

251 926 individuals

with at least one MI

243 091 individuals

with a first MI

242 665 individuals

with valid data

426 individuals with a mismatch between their SMR01 and death records

8835 individuals with an MI recorded in the previous 10 years

5619 individuals with a history of another mental health condition^1^

237 046 individuals with no history of a mental health condition or with a history of schizophrenia, bipolar disorder, depression

1736 individuals with missing data for area-based deprivation, urbanity or health board

235 310 individuals

in the cohort

226 537 individuals with first MI between 1991 and 2013^2^

188 930 individuals who survived more than 30 days^3^

1. Including other psychoses, other mood disorders, disorders of adult personality and behaviour, eating disorders, neuroses, dissociative and somatoform disorders, behavioural and emotional disorders with onset in childhood and adolescence, non-organic sleep disorders, disorders of psychosocial development and unspecified mental disorders.
2. Restricted cohort for the analysis of 5-year mortality.
3. Restricted cohort for the analysis of time to further event outcomes.

# Table S5: Number of individuals and events per group

| Outcome | No mental health admission | Schizophrenia | Bipolar disorder | Depression | Total |
| --- | --- | --- | --- | --- | --- |
| Complete cases | 227 506 | 923 | 642 | 6239 | 235 310 |
| 30-day mortality | 44 580 (19.6%) | 203 (22.0%) | 154 (24.0%) | 1443 (23.1%) | 46 380 (19.7%) |
| 1-year mortality | 66 977 (29.4%) | 309 (33.5%) | 247 (38.5%) | 2306 (37.0%) | 69 839 (29.7%) |
| Mortality during follow-up | 160 716 (70.6%) | 684 (74.1%) | 487 (75.9%) | 4658 (74.7%) | 166 545 (70.8%) |
| Revascularisation within 30 days | 37 375 (16.4%) | 137 (14.8%) | 94 (14.6%) | 1036 (16.6%) | 38 642 (16.4%) |
| Revascularisation within 90 days | 41490 (18.2%) | 142 (15.4%) | 104 (16.2%) | 1126 (18.0%) | 42862 (18.2%) |
| Complete cases  (MI admissions up to 2013) | 219 161 | 868 | 611 | 5897 | 226 537 |
| 5-year mortality | 100 560 (45.9%) | 457 (52.6%) | 350 (57.3%) | 3388 (57.5%) | 104 755 (46.2%) |
| Complete cases  (individuals who survived  more than 30 days) | 182 926 | 720 | 488 | 4796 | 188 930 |
| Time to further MI | 53 376 (29.2%) | 205 (28.5%) | 139 (28.5%) | 1496 (31.2%) | 55 216 (29.2%) |
| Time to further vascular event | 65 857 (36.0%) | 248 (34.4%) | 176 (36.1%) | 1845 (38.5%) | 68 126 (36.1%) |

# Table S6: Number of individuals and events per group – sensitivity analysis (depression based on psychiatric hospital admission records only)

| Outcome | No mental health admission | Schizophrenia | Bipolar disorder | Depression | Total |
| --- | --- | --- | --- | --- | --- |
| Complete cases | 230 048 | 923 | 642 | 3111 | 234 724 |
| 30-day mortality | 45 249 (19.7%) | 203 (22.0%) | 154 (24.0%) | 663 (21.3%) | 46 269 (19.7%) |
| 1-year mortality | 68 044 (29.6%) | 309 (33.5%) | 247 (38.5%) | 1033 (33.2%) | 69 633 (29.7%) |
| Mortality during follow-up | 162 709 (70.7%) | 684 (74.1%) | 487 (75.9%) | 2259 (72.6%) | 166 139 (70.8%) |
| Revascularisation within 30 days | 37 741 (16.4%) | 137 (14.8%) | 94 (14.6%) | 548 (17.6%) | 38 520 (16.4%) |
| Revascularisation within 90 days | 41 893 (18.2%) | 142 (15.4%) | 104 (16.2%) | 599 (19.3%) | 42 738 (18.2%) |
| Complete cases  (MI admissions up to 2013) | 221 553 | 868 | 611 | 2958 | 225 990 |
| 5-year mortality | 102 103 (46.1%) | 457 (52.6%) | 350 (57.3%) | 1544 (52.2%) | 104 454 (46.2%) |
| Complete cases  (individuals who survived  more than 30 days) | 184 799 | 720 | 488 | 2448 | 188 455 |
| Time to further MI | 53 970 (29.2%) | 205 (28.5%) | 139 (28.5%) | 756 (30.9%) | 55 070 (29.2%) |
| Time to further vascular event | 66 593 (36.0%) | 248 (34.4%) | 176 (36.1%) | 939 (38.4%) | 67 956 (36.1%) |

# Table S7: Sensitivity analysis for models 1 and 2 (depression based on psychiatric hospital admission records only)

For each outcome, this table presents a summary of the results for models 1 and 2, along with a summary of the results of the sensitivity analysis for models 1 and 2. In the sensitivity analysis, depression is only identified using psychiatric hospital admission records (SMR04). Thus fewer people are included in the depression group, and the overall cohort is smaller. The results for schizophrenia and bipolar disorder differ slightly between the main analysis and the sensitivity analysis because the comparison group has changed (some people who were included in the depression group for the main analysis are included in the no mental health admission group for the sensitivity analysis).

| Outcome | Model | N | Schizophrenia | Bipolar disorder | Depression |
| --- | --- | --- | --- | --- | --- |
| 30-day mortality, OR (95% CI) | Model 1 | 235 310 | 2.06 (1.74, 2.44) | 1.58 (1.30, 1.91) | 1.37 (1.29, 1.46) |
|  | Model 1 (depression – SMR04 only) | 234 724 | 2.05 (1.73, 2.43) | 1.57 (1.29, 1.90) | 1.36 (1.24, 1.49) |
|  | Model 2 | 235 310 | 1.95 (1.64, 2.30) | 1.53 (1.26, 1.86) | 1.31 (1.23, 1.40) |
|  | Model 2 (depression – SMR04 only) | 234 724 | 1.94 (1.63, 2.29) | 1.52 (1.25, 1.84) | 1.27 (1.16, 1.40) |
| 1-year mortality, OR (95% CI) | Model 1 | 235 310 | 2.41 (2.07, 2.81) | 1.99 (1.66, 2.37) | 1.65 (1.55, 1.75) |
|  | Model 1 (depression – SMR04 only) | 234 724 | 2.40 (2.05, 2.79) | 1.97 (1.65, 2.35) | 1.54 (1.42, 1.68) |
|  | Model 2 | 235 310 | 2.22 (1.91, 2.59) | 1.90 (1.59, 2.27) | 1.53 (1.45, 1.63) |
|  | Model 2 (depression – SMR04 only) | 234 724 | 2.20 (1.89, 2.56) | 1.88 (1.57, 2.25) | 1.40 (1.29, 1.52) |
| 5-year mortality, OR (95% CI) | Model 1 | 226 537^a^ | 3.11 (2.67, 3.63) | 2.28 (1.89, 2.75) | 2.08 (1.96, 2.22) |
|  | Model 1 (depression – SMR04 only) | 225 990^a^ | 3.08 (2.64, 3.59) | 2.26 (1.87, 2.72) | 1.84 (1.69, 2.01) |
|  | Model 2 | 226 537 ^a^ | 2.75 (2.35, 3.20) | 2.15 (1.78, 2.59) | 1.82 (1.71, 1.94) |
|  | Model 2 (depression – SMR04 only) | 225 990 ^a^ | 2.70 (2.32, 3.15) | 2.12 (1.76, 2.55) | 1.55 (1.43, 1.69) |
| Mortality during follow-up, HR (95% CI) | Model 1 | 235 310 | 1.97 (1.82, 2.12) | 1.63 (1.50, 1.79) | 1.44 (1.40, 1.49) |
|  | Model 1 (depression – SMR04 only) | 234 724 | 1.96 (1.82, 2.11) | 1.63 (1.49, 1.78) | 1.40 (1.34, 1.46) |
|  | Model 2 | 235 310 | 1.82 (1.68, 1.96) | 1.55 (1.42, 1.70) | 1.35 (1.31, 1.39) |
|  | Model 2 (depression – SMR04 only) | 234 724 | 1.80 (1.67, 1.94) | 1.54 (1.41, 1.68) | 1.27 (1.22, 1.32) |
| Time to further MI,  HR (95% CI) | Model 1 | 188 930^b^ | 1.50 (1.31, 1.72) | 1.38 (1.17, 1.63) | 1.45 (1.38, 1.53) |
|  | Model 1 (depression – SMR04 only) | 188 455 ^b^ | 1.50 (1.30, 1.72) | 1.38 (1.16, 1.63) | 1.38 (1.29, 1.49) |
|  | Model 2 | 188 930 ^b^ | 1.42 (1.24, 1.63) | 1.34 (1.13, 1.58) | 1.38 (1.31, 1.45) |
|  | Model 2 (depression – SMR04 only) | 188 455 ^b^ | 1.41 (1.23, 1.62) | 1.33 (1.12, 1.57) | 1.29 (1.20, 1.39) |
| Time to further MI or stroke  HR (95% CI) | Model 1 | 188 930 ^b^ | 1.55 (1.37, 1.75) | 1.45 (1.25, 1.68) | 1.48 (1.41, 1.55) |
|  | Model 1 (depression – SMR04 only) | 188 455 ^b^ | 1.54 (1.36, 1.75) | 1.44 (1.24, 1.67) | 1.42 (1.33, 1.51) |
|  | Model 2 | 188 930 ^b^ | 1.46 (1.29, 1.65) | 1.40 (1.20, 1.62) | 1.40 (1.33, 1.46) |
|  | Model 2 (depression – SMR04 only) | 188 455 ^b^ | 1.45 (1.28, 1.64) | 1.39 (1.19, 1.61) | 1.31 (1.23, 1.40) |
| Revascularisation within 30 days, HR (95% CI) | Model 1 | 235 310 | 0.52 (0.44, 0.62) | 0.66 (0.54, 0.80) | 0.71 (0.67, 0.76) |
|  | Model 1 (depression – SMR04 only) | 234 724 | 0.53 (0.45, 0.62) | 0.66 (0.54, 0.81) | 0.81 (0.75, 0.88) |
|  | Model 2 | 235 310 | 0.57 (0.48, 0.67) | 0.69 (0.56, 0.85) | 0.78 (0.73, 0.83) |
|  | Model 2 (depression – SMR04 only) | 234 724 | 0.58 (0.49, 0.68) | 0.70 (0.57, 0.85) | 0.90 (0.82, 0.98) |
| Revascularisation within 90 days, HR (95% CI) | Model 1 | 235 310 | 0.49 (0.42, 0.58) | 0.66 (0.54, 0.80) | 0.70 (0.66, 0.75) |
|  | Model 1 (depression – SMR04 only) | 234 724 | 0.49 (0.42, 0.58) | 0.66 (0.55, 0.81) | 0.81 (0.74, 0.87) |
|  | Model 2 | 235 310 | 0.53 (0.45, 0.63) | 0.69 (0.57, 0.84) | 0.77 (0.73, 0.82) |
|  | Model 2 (depression – SMR04 only) | 234 724 | 0.54 (0.46, 0.63) | 0.70 (0.58, 0.85) | 0.89 (0.82, 0.97) |

Model 1 is adjusted for age at MI, sex and year of MI. Model 2 is adjusted for age at MI, sex, year of MI, history of alcohol use disorder, deprivation, urbanity and health board. In the sensitivity analysis, depression is only identified using mental health hospital admission records (SMR04).

HR=Hazard ratio. OR=Odds ratio.

a Stroke admissions up to 2013 in order to ensure that all individuals have at least 5 years’ follow-up.

b Individuals who survived more than 30 days after MI.

# Table S8: P-values for analysis of deviance comparing models without each interaction to the model including all three interactions

|  |  | Interaction | | |
| --- | --- | --- | --- | --- |
| Outcome | Model | SMI: age at MI | SMI: year of MI | SMI: sex |
| 30-day mortality | Model 3 | <0.0001 | 0.64 | 0.19 |
|  | Model 3 (depression - SMR04 only) | 0.0029 | 0.65 | 0.59 |
| 1-year mortality | Model 3 | 0.00014 | 0.18 | 0.38 |
|  | Model 3 (depression - SMR04 only) | 0.0037 | 0.58 | 0.63 |
| 5-year mortality | Model 3 | 0.15 | 0.59 | 0.30 |
|  | Model 3 (depression - SMR04 only) | 0.28 | 0.83 | 0.24 |
| Mortality during follow-up | Model 3 | <0.0001 | <0.0001 | 0.094 |
|  | Model 3 (depression - SMR04 only) | <0.0001 | 0.12 | 0.077 |
| Time to further myocardial infarction | Model 3 | 0.87 | 0.16 | 0.25 |
|  | Model 3 (depression - SMR04 only) | 0.78 | 0.45 | 0.27 |
| Time to further vascular event | Model 3 | 0.31 | 0.018 | 0.29 |
|  | Model 3 (depression - SMR04 only) | 0.29 | 0.29 | 0.19 |
| Revascularisation within 30 days | Model 3 | <0.0001 | 0.19 | 0.00031 |
|  | Model 3 (depression - SMR04 only) | 0.00032 | 0.31 | 0.00063 |
| Revascularisation within 90 days | Model 3 | <0.0001 | 0.062 | <0.0001 |
|  | Model 3 (depression - SMR04 only) | 0.00017 | 0.33 | 0.00012 |

# Figure S2: Odds ratios for 5-year mortality comparing people with a hospital record for each SMI versus no record of any mental health condition

Sex-specific odds ratios and 95% confidence intervals for 5-year mortality following a myocardial infarction, among 70-year olds, comparing people with a prior hospital record for each SMI versus no prior record of any mental health condition, 1991 – 2013. Estimates were obtained from a logistic regression model adjusting for age at MI, year of MI, sex, deprivation, urbanity, health board and history of an alcohol use disorder, and including interactions between SMI and each of age at MI, year of MI and sex. Shading represents 95% confidence intervals.


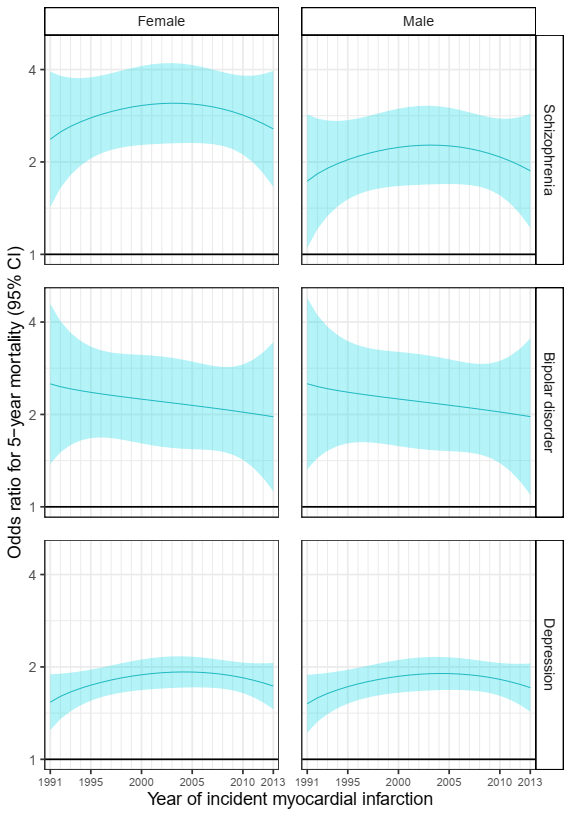


# Figure S3: Hazard ratios for mortality during follow-up comparing people with a hospital record for each SMI versus no record of any mental health condition

Sex-specific hazard ratios and 95% confidence intervals for morality during follow-up after a myocardial infarction, among 70-year olds, comparing people with a prior hospital record for each SMI versus no prior record of any mental health condition, 1991 – 2014. Estimates were obtained from a Cox proportional hazards model adjusting for age at MI, year of MI, sex, deprivation, urbanity, health board and history of an alcohol use disorder, and including interactions between mental health condition and each of age at MI, year of MI and sex. Shading represents 95% confidence intervals.


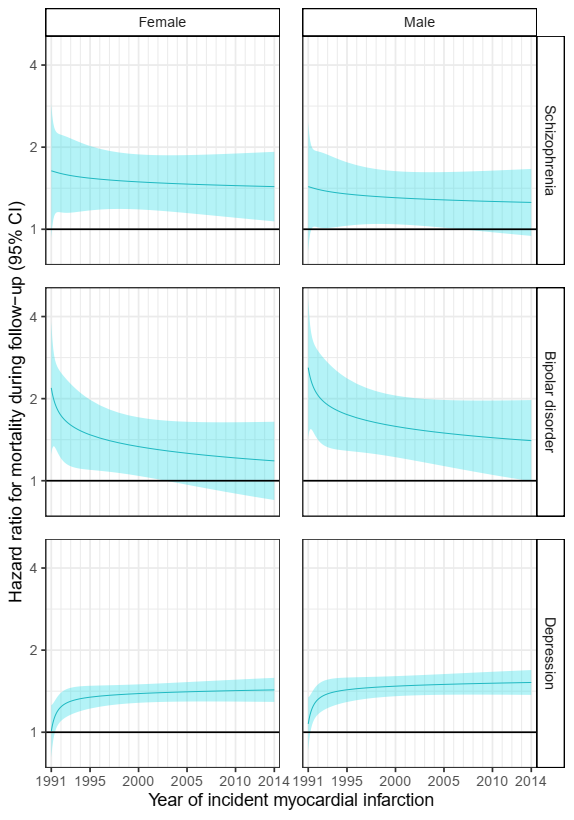


# Figure S4: Hazard ratios for time to further myocardial infarction comparing people with a hospital record for each SMI versus no record of any mental health condition

Sex-specific hazard ratios and 95% confidence intervals for time to further myocardial infarction following first myocardial infarction, among 70-year olds, comparing people with a hospital record for each SMI versus no record of any mental health condition, 1991 - 2014. Estimates were obtained from a Cox proportional hazards model adjusting for age at MI, year of MI, sex, deprivation, urbanity, health board and history of an alcohol use disorder, and including interactions between SMI and each of age at MI, year of MI and sex. Shading represents 95% confidence intervals.


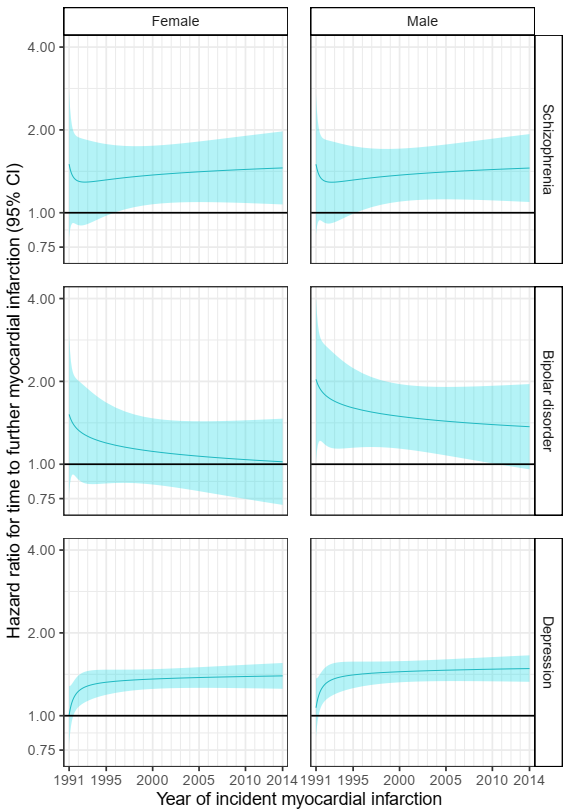


# Figure S5: Hazard ratios for time to further vascular event (MI or stroke) comparing people with a hospital record for each SMI versus no record of any mental health condition

Sex-specific hazard ratios and 95% confidence intervals for time to further vascular event (MI or stroke) following first myocardial infarction, among 70-year olds, comparing people with a hospital record for each SMI versus no record of any mental health condition, 1991 to 2014. Estimates were obtained from a Cox proportional hazards model adjusting for age at MI, year of MI, sex, deprivation, urbanity, health board and history of an alcohol use disorder, and including interactions between SMI and each of age at MI, year of MI and sex. Shading represents 95% confidence intervals.


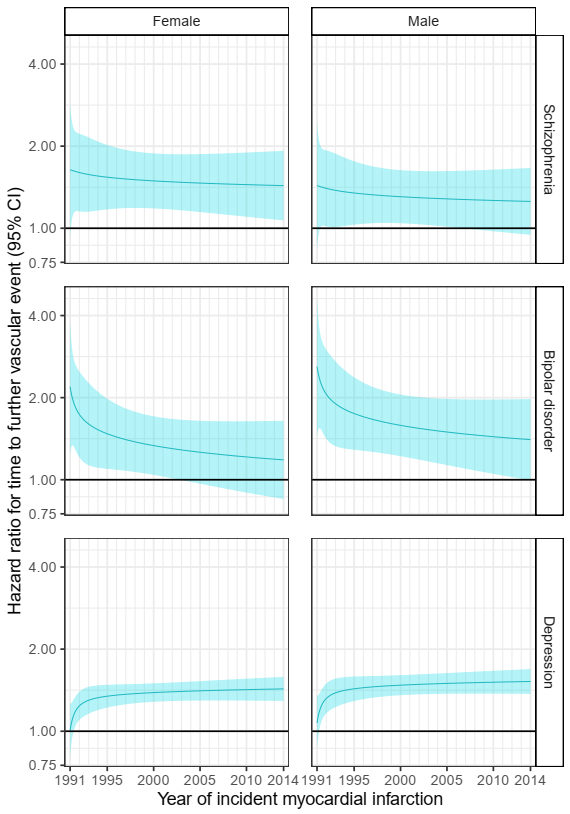


# Figure S6: Hazard ratios for revascularisation within 90 days comparing people with a hospital record for each SMI versus no record of any mental health condition

Sex-specific hazard ratios and 95% confidence intervals for revascularisation within 90 days following a myocardial infarction, among 70-year olds, comparing people with a hospital record for each SMI versus no record of any mental health condition, 1991 - 2014. Estimates were obtained from a Cox proportional hazards model adjusting for age at MI, year of MI, sex, deprivation, urbanity, health board and history of an alcohol use disorder, and including interactions between SMI and each of age at MI, year of MI and sex. Shading represents 95% confidence intervals.


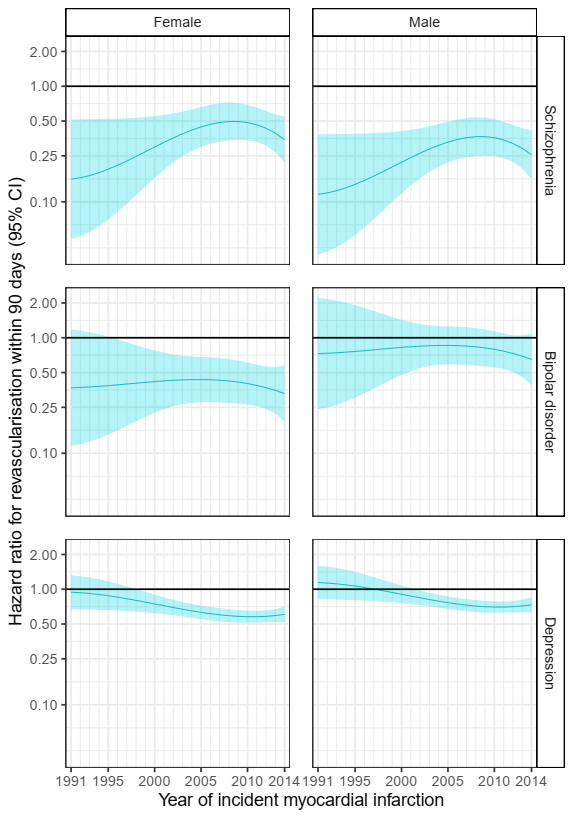

Supplement: Supplementary file 1 — Additional file 1: : Table S1. ICD-9 and ICD-10 codes used to identify mental health conditions. Table S2. OPCS-4 codes used to identify coronary revascularisation procedures. Text S1. Carstairs Index. Table S3. ICD-9 and ICD-10 codes used to identify alcohol use disorder. Table S4. ICD codes used to identify comorbidities recorded during the incident MI admission. Fig. S1. Flow diagram for establishing the cohort. Table S5. Number of individuals and events per group. Table S6. Number of individuals and events per group – sensitivity analysis (depression based on psychiatric hospital admission records only). Table S7. Sensitivity analysis for models 1 and 2 (depression based on psychiatric hospital admission records only). Table S8. P-values for analysis of deviance comparing models without each interaction to the model including all three interactions. Fig. S2. Odds ratios for 5-year mortality comparing people with a hospital record for each SMI versus no record of any mental health condition. Fig. S3. Hazard ratios for mortality during follow-up comparing people with a hospital record for each SMI versus no record of any mental health condition. Fig. S4. Hazard ratios for time to further myocardial infarction comparing people with a hospital record for each SMI versus no record of any mental health condition. Fig. S5. Hazard ratios for time to further vascular event (MI or stroke) comparing people with a hospital record for each SMI versus no record of any mental health condition. Fig. S6. Hazard ratios for revascularisation within 90 days comparing people with a hospital record for each SMI versus no record of any mental health condition. [file 12916_2021_1937_MOESM1_ESM.docx]
